# Estimating the returns to UK publicly funded cancer-related research in terms of the net value of improved health outcomes

**DOI:** 10.1186/1741-7015-12-99

**Published:** 2014-06-16

**Authors:** Matthew Glover, Martin Buxton, Susan Guthrie, Stephen Hanney, Alexandra Pollitt, Jonathan Grant

**Affiliations:** 1 Health Economics Research Group, Brunel University, Uxbridge, UK; 2 RAND Europe, Westbrook Centre, Milton Road, Cambridge CB4 1YG, UK; 3 King’s Policy Institute, King’s College London, Virginia Woolf Building, 22 Kingsway, London WC2R 2LA, UK

**Keywords:** Medical research investment, QALYs, Cancer, Medical research charities, Value of health, Rate of return, Time lags, Research payback

## Abstract

**Background:**

Building on an approach developed to assess the economic returns to cardiovascular research, we estimated the economic returns from UK public and charitable funded cancer-related research that arise from the net value of the improved health outcomes.

**Methods:**

To assess these economic returns from cancer-related research in the UK we estimated: 1) public and charitable expenditure on cancer-related research in the UK from 1970 to 2009; 2) net monetary benefit (NMB), that is, the health benefit measured in quality adjusted life years (QALYs) valued in monetary terms (using a base-case value of a QALY of GB£25,000) minus the cost of delivering that benefit, for a prioritised list of interventions from 1991 to 2010; 3) the proportion of NMB attributable to UK research; 4) the elapsed time between research funding and health gain; and 5) the internal rate of return (IRR) from cancer-related research investments on health benefits. We analysed the uncertainties in the IRR estimate using sensitivity analyses to illustrate the effect of some key parameters.

**Results:**

In 2011/12 prices, total expenditure on cancer-related research from 1970 to 2009 was £15 billion. The NMB of the 5.9 million QALYs gained from the prioritised interventions from 1991 to 2010 was £124 billion. Calculation of the IRR incorporated an estimated elapsed time of 15 years. We related 17% of the annual NMB estimated to be attributable to UK research (for each of the 20 years 1991 to 2010) to 20 years of research investment 15 years earlier (that is, for 1976 to 1995). This produced a best-estimate IRR of 10%, compared with 9% previously estimated for cardiovascular disease research. The sensitivity analysis demonstrated the importance of smoking reduction as a major source of improved cancer-related health outcomes.

**Conclusions:**

We have demonstrated a substantive IRR from net health gain to public and charitable funding of cancer-related research in the UK, and further validated the approach that we originally used in assessing the returns from cardiovascular research. In doing so, we have highlighted a number of weaknesses and key assumptions that need strengthening in further investigations. Nevertheless, these cautious estimates demonstrate that the returns from past cancer research have been substantial, and justify the investments made during the period 1976 to 1995.

## Background

### Estimating the returns from biomedical and health research

Estimating the economic returns arising from health research develops our understanding of how research translates from ‘bench to bedside’, can be used in advocating the case for future investments in medical research, and demonstrates accountability for public and charitable research funding to taxpayers and donors. Because resources used for publicly and charitably funded medical research, including cancer research, could potentially be put to other purposes for the benefit of society, there is an obligation to demonstrate that such investments represent good value. In the medical field, it is possible to identify illustrative examples of specific research breakthroughs that have contributed to substantial benefit in terms of life-saving interventions, or to major improvements in the quality of life of patients with a chronic disease. However, it is much more difficult to describe systematically the nature and extent of the returns to the investment of a whole body of medical research, some of which may inevitably be less fruitful. Furthermore, there are tensions between advocacy, where interested parties are arguing for more research funding, and more dispassionate analysis, which might conclude that too much money is being spent on research. As noted in an editorial in *Nature* in 2010: ‘Most of the attempts to count the economic benefits of investment in science have been derived from the efforts of lobbying groups and funding agencies to justify science spending’ [1].

The literature that assesses the value of the benefits of medical research forms a relatively small field in terms of methodology and quality [2,3]. There is a lack of clear consensus about key issues, such as the best methods to use to assess the value of the health gains, and there is also variability in the extent to which studies have included all the important components required for a full analysis of the cost-effectiveness of investing in research. As summarised in Table 1, Mushkin [4], in an early study, used a human capital approach to value health gains from all US biomedical research in terms of the productivity gains from having a healthy workforce [4]. This approach has various weaknesses, which were recognised by Mushkin and others [5], including that it tends to overstate the benefits when lost labour can be replaced by unemployed people or through migration, and it undervalues health gains for groups such as the elderly. Funding First [6] advanced the field by building on a different approach based on estimates of the average willingness of individuals to pay for small reductions in the risk of death. They used this figure to value the increased longevity of the US population. In a background paper for this, Murphy and Topel [7] calculated the enormous economic value that would come from finding a cure for cancer and other diseases, but to date, and using the methods they had adopted, the Funding First report claimed ‘the largest returns to investment in medical research have come principally from gains against heart disease and stroke’ ([6], page 3).

**Table 1:** Methods used in various studies to assess the benefits from health research

**Study/features**	**Mushkin (1979) [**[4]**]**	**Funding first (2000) [**[6]**]**	**Access economics (2003) [**[8]**]**	**Access economics (2008) [**[9]**]**	**HERG *et al. *** **(2008) [**[3]**]**	**Access economics (2011) [**[10]**]**
How health gains were assessed	Top-down by disease category: overall gain in each category not linked to specific intervention. Attributed 20 to 30% of total gain to R&D. Reduced morbidity difficult to assess because little reduction in days off work because of sickness. Adjusted the raw data, for example, by applying historical Army and Navy data as an index to record the decline in sickness.	Top-down: overall gain in mortality not linked to specific interventions. Attributed roughly one-third of the total gain to R&D, plus ‘some fraction of the credit for the other two-thirds.’	Top-down: overall gain in mortality and morbidity not linked to specific interventions. Attributed 50% of the total gain to R&D.	Top-down: as in the 2003 study, overall gain in mortality and morbidity not linked to specific interventions. Attributed 50% of the total gain to R&D.	Bottom-up: identified research-based interventions, then quantified health impact.	Top-down: overall gain in mortality and morbidity for five disease areas not linked to specific interventions. Attributed 50% of the total gain to R&D.
How health gains were valued	Human capital approach, that is, values attached to lives saved between one period and the next, based on potential future earnings, plus calculation of value of potential working time no longer lost due to sickness.	Used a comparatively high ‘willingness-to-pay’ value derived from labour economics.	Used the same comparatively high ‘willingness-to-pay’ value as Funding First.	Used a higher ‘willingness-to-pay’ estimate than the 2003 study, this time derived from a meta-analysis of international studies.	Used a comparatively low, but arguably realistic, value of health gain by adopting the figure implied by the current level of NHS spending, that is, the opportunity cost of a QALY within the current NHS budget.	Used a lower ‘willingness-to-pay’ estimate than that used in the 2008 study, in line with Department of Finance and Deregulation guidance.
Proportion of national health gain allocated to national research	Not discussed as a major issue; we assumed it to be 100%.	Not discussed as a major issue in Funding First; we assumed it to be 100%.	Used proportion of global research conducted in Australia (2.5%) to determine the proportion of the total research-based health gain to attribute to Australian research.	Uses bibliometric analysis-based estimate of Australian share of global research output in clinical medicine (3.04%).	An analysis of citations of UK research on UK clinical guidelines suggests average best estimate of 17% linked to UK research.	Uses an updated bibliometric analysis-based estimate of Australian share of global research output in clinical medicine (3.14%).
Costs of health care considered?	No, at least not as a separate item to net-off against the value of the health gains.	No in initial headline figures, but Yes in later analysis: ‘the gain in the value of life, net what was spent to attain the longer life, is just 15 percent smaller.’	No, did not net-off the healthcare costs required to achieve the health gains.	No, did not net-off the healthcare costs required to achieve the health gains.	Yes, did net-off the health care costs required to achieve the health gains.	Did not net-off health care delivery costs, but did consider avoided health system expenditure due to gains in wellbeing.
Considered elapsed time between research and health gains?	Yes: 10 years.	Acknowledged time lags between research and benefits but this was apparently not brought into calculations.	No, compared research expenditure and health benefits in the same year. This implies the health gains from research are instant.	Yes: 40 years, with range of 20 to 60 years used for sensitivity analyses.	Yes: an analysis of citations of UK research on UK clinical guidelines suggested average best estimate of 17 years lag.	Yes: same assumption of 40 years as was used in 2008 study. No sensitivity analysis around elapsed time.
How the overall rate of return calculated	IRR of 47%.	Not brought together to provide an overall IRR.	An overall benefit/costs ratio for health research of 2.40.	An overall benefit/costs ratio for health research of 2.17.	IRR of 9% for CVD research combined with 30% for GDP benefits.	Benefit-cost ratios for five disease areas: 6.1 (CVD); 2.7 (cancer); 1.1 (SIDS); 1.2 (asthma); and 0.7 (muscular dystrophy).

*Abbreviations:CVD* cardiovascular disease, *GDP* Gross Domestic Product, *IRR* internal rate of return, *NHS* National Health Service, *QALY* quality adjusted life years, *R&D* research and development, *SIDS* Sudden Infant Death Syndrome.

A broadly similar approach was adopted in a series of Australian studies conducted by Access Economics (2003, 2008 and 2011) [8-10], but expanded to allow for improvements in quality of life based on disability adjusted life years (DALYs). In the 2003 version of the report, no allowance was made for the elapsed time between research (input) and improved health and wellbeing (outcome). In the 2008 and 2011 iterations, this was addressed by projecting potential health and wellbeing gains 40 years into the future. In the 2011 report, the authors focused on estimating a return on investment for five specific diseases, including cancer.

To date, only three studies that we are aware of have examined the economic returns from cancer research. Two of those focused on the costs and benefits of US President Nixon’s ‘War on Cancer’ [11-13]. Litchenberg [11] in 2004 examined the contribution of pharmaceutical innovation to increases in cancer survival rates, by looking at the number of new drugs that had been approved to treat cancer after 1971 (when the War on Cancer was declared), and modelling the impact on cancer mortality rates in the US. He estimated that the increase in approved drugs accounted for about 50 to 60% of the increase in age-adjusted cancer survival rates. Although Litchenberg [11] did not compute a rate of return, he did note that the drug costs to achieve an additional year of life per person diagnosed with cancer were well below estimates for the value of a statistical life. Pertinent to the approach adopted in the current study, he concluded: ‘Ideally, we would have measured the effects of new cancer drugs on the number of quality adjusted life years (QALYS), but were unable to do so due to lack of data’. In two related papers, Sun *et al*. [12] and Lakdawalla *et al*. [13] followed a similar conceptual approach in quantifying the value of gains in cancer survival, but directly compared this with the costs of research and development (R&D). They estimated that improvements in cancer survival in the US between 1988 and 2000 created 23 million additional life years, equivalent to roughly US$1.9 trillion of additional social value, implying that the average life year gained was worth US$82,000. As with Litchenberg [11], Sun *et al*. did not calculate a return on investment but noted that ‘These calculations suggest that from the patient’s point of view, the rate of return to R&D investments against cancer has been substantial.’ The third study to look explicitly at cancer is the Deloitte Access Economics study [10] cited above. In that report, the authors looked at the rate of return from current (2000 to 2010) research investment in cancer by the Australian National Health and Medical Research Council (NHMRC) and compared this with gains in wellbeing using DALYS projected for 2040 to 2050. In doing so, they estimated the net benefit of NHMRC R&D between 2000 and 2010 to be AU$1.96 billion with a cost/benefit ratio of 2.7; that is, for every AU$1 million invested in cancer research they would anticipate a return worth $1.7 million.

A recurring theme in these studies is the extent to which health gains can be attributed to research-inspired medical advances. Funding First and Access Economics adopted a ‘top-down’ (or macro) approach that took a measure of the overall national health gain from various fields of medicine, and then assumed that a proportion was attributable to medical research. One way of addressing this problem of attribution is by examining in a bottom-up manner the impacts of specific projects or programmes of research by tracing forwards from the research to the benefits that arise. Here, considerable progress has been made using the Payback Framework [14-18], but this has relied on the development of specific resource-intensive case studies. Other studies have made progress in analysing the value of the health gains associated with a series of clinical trials [19], but the major challenge faced by these types of studies is attribution: that is, how to show that the health gains that have arisen can be attributed to specific pieces of research.

In 2008, we published a report, funded by the Wellcome Trust and UK Medical Research Council, which aimed to build on the advances in previous studies and address the existing limitations, so as to develop an approach that could be used to measure the economic benefits accruing from publicly and charitably funded medical research [3]. We analysed two major elements of economic returns: the broad impact on the UK Gross Domestic Product (GDP) and the specific net monetary benefits (NMB), defined as the health benefit valued in monetary terms minus the cost of delivering that health benefit which arose from the UK application of relevant UK research. Our analysis of the existing evidence on the GDP or ‘spillover’ benefits, based largely on US studies from a number of areas of research and certainly not specific to any particular area of medical research, suggested a best estimate of an internal rate of return (IRR) of around 30%. We estimated the NMB of the health gain using methods similar to those used here, giving an IRR of 9% for cardiovascular research. This meant that a GB£1.00 investment in publicly/charitably funded CVD research produced a combined stream of benefits thereafter, equivalent in value to earning £0.39 per year in perpetuity. (We also estimated the NMB from mental health research, which produced an IRR of 7%; however, this was based on a more limited analysis because of data limitation and uncertainties around the effects of interventions in mental health, which meant that we were less confident in the results than we were for the CVD results).

These estimates of the IRR have been widely used in policy circles in the UK and beyond [20-23], and in the absence of any other estimates of the economic impact of biomedical research, the figures have often been used as proxies of the economic impact of medical research more broadly. A consortium of funders (Wellcome Trust, National Institute of Health Research, Cancer Research UK (CRUK), and the Academy of Medical Sciences) commissioned a study to further validate the approach and to explore whether the IRR from the net value of the health benefits in another area, cancer, was similar or not. Thus, this study aimed to estimate the economic returns from UK publicly and charitably funded cancer research on improved health outcomes in the UK specifically. As with the previous CVD study, we accept that there are international benefits of UK research, but this was not in the scope of the current exercise, although as we note, this is an area that warrants further investigation. In addition, and as reported separately [24], we undertook five exploratory case studies to understand qualitatively the complexity of how research translates into health benefit.

We present the methods used for the four main steps that provided the estimated parameters to enable us to calculate the economic returns from the NMB of the UK health gains that we attributed to past UK publicly and charitably funded cancer-related research, and present the results expressed as estimates of the IRR, with sensitivity analyses to illustrate the effects of some of the key uncertainties. Finally, we explored the significance of our findings in the context of previous studies and the wider policy debate on R&D investments and economic impact; we detailed the limitations of our approach; and we developed a research agenda for this fledgling field.

## Methods

### Overall conceptual approach

Four key sources of data were needed to estimate the IRR of the NMB of the health gains arising from cancer research:

• a time series of the public and charitable funding of cancer-related research;

• a time series of the NMB of cancer health gains, derived from the monetised health benefits and the healthcare costs for selected interventions^a^;

• an estimate of the elapsed time between the investment (research funding) and return (health gain) associated with those interventions; and

• an estimate of the amount of health gain that should be attributed to public and charitable research investment in cancer-related research in the UK.

With these four data inputs, we then calculated a rate of return on the investment in cancer research.

It should be noted that the costs of private sector R&D investments are accounted for in our analysis as elements within the cost of delivering health care, which are netted off in the NMB. The costs to the health service of medical interventions produced by the private sector include the return to the private sector on its R&D investments.

### Estimating public and charitable funding of cancer-related research

The leading funders of cancer research in the UK were identified by examining the National Cancer Research Institute (NCRI) Cancer Research Database. Between 2002 and 2011, the top 10 funders consistently accounted for over 95% of cancer research spend by the 21 NCRI partners.^b^ Estimates of annual cancer-related research funding between 1970 and 2009 were assembled for these 10 organisations plus an estimated contribution to cover Funding Council support for cancer research (the Higher Education Funding Council for England and similar bodies in Wales, Scotland and Northern Ireland provide a performance-related block grant to UK universities based on the quality and volume of research). A detailed account of how we estimated these 11 time series is provided (see Additional file 1).

As also discussed in detail in Additional file 1, in estimating research spend for the Funding Councils and the Department of Health (DH)/NHS, we had to derive a figure specifically for cancer-related research activity in the UK. We settled on a central estimate of 10% of total publicly and charitably funded health and biomedical research activity, and we also assumed it to be constant over the time period. This estimate was derived from a number of independent sources, as follows

• Medical Research Council (MRC) spending on cancer research averaged 9.8% of their total investment (range: 4.6% to 16.7%) between 1970/1 and 2009/10.

• Wellcome Trust cancer funding was more erratic, ranging between 1%^c^ and 38%, with an average of 14.5% of expenditure being on cancer research.

• The proportion of peer-reviewed research papers in oncology as a percentage of all UK biomedical outputs averaged 9.2% (range: 8.5% to 9.5%) between 1988 and 1995 [25].

• The proportion of peer-reviewed research papers in oncology research (as a percentage of all NHS research outputs) was 12% between 1990 and 1997 [26].

• The proportion of mainstream quality-related (QR) funding allocations by the Higher Education Funding Council for England for ‘Cancer studies’ (that is, Unit of Assessment 02) between 2009 and 2012 was around 6% of the total biomedical allocation (that is, Unit of Assessments 01 to 15 and 44).^d^

Given the importance of this estimate of 10% for the proportion of research activity that is related to cancer (for those sources where we had no actual breakdown), we also looked at the effect of lower and higher estimates of 7.5% and 15%, respectively, in the sensitivity analyses.

### Estimating the NMB from cancer-related research

This element of the research required estimates of the lifetime QALYs gained and the net lifetime costs to the NHS of delivering those QALYs for research-based interventions provided in each year of the period 1991 to 2010. The general methods mirrored those used in the 2008 study [3] on the returns on investment in CVD research, and again built up the aggregate net benefits from the bottom up, aggregating the QALYs gained and the net NHS costs from the use of specific interventions. This approach required: 1) identification of the key relevant cancer interventions and their level of usage during the relevant period; and 2) estimates of the QALY gains and NHS costs associated with the interventions. From this information, the NMB was calculated as the health benefit valued in monetary terms (determined by the quantity of health benefit and a decision-maker’s willingness to pay for that additional benefit) minus the cost of delivering that health benefit.

In the CVD study, our starting point was previously published research identifying the cardiovascular interventions that had contributed most health gain [27]. No equivalent studies for cancer were identified that could provide a comparable basis for deciding which interventions were, quantitatively, the most important to include in the analysis. Thus, the three main steps for quantifying the total NMB associated with cancer interventions were: 1) to identify the cancer interventions that were the likely major sources of benefits; 2) to identify appropriate estimates of NMB per patient for that subset of cancer interventions; and 3) to construct a time series (for 1991 to 2010) of the number of patients receiving each of these subsets of cancer intervention in the UK.

### Identifying the key cancer interventions

At the outset of the study, we had a number of discussions with cancer research experts to provide us with a broad understanding of the main developments in the field over the past 20 years. Informed by these discussions, we quantitatively identified those areas that had resulted in the largest health gain in the UK since 1990, arising from three main sources: 1) key cancers where research and resultant health policies have led to health gains through a reduction in incidence; 2) key cancers for which screening programmes have led to health gains because of early detection; and 3) key cancers where there have been the most significant health gains from increased survival.

To identify areas where a reduction in incidence has been observed, cancer incidence data in the UK were analysed, using UK incidence rates between 1990 and 2008 [28], to calculate a percentage change over the period. This percentage change was then multiplied by mid-period UK incidence (the average per year for 1999 to 2001 [29]) to estimate an absolute change in incidence. Four cancer types have seen significantly larger reductions in incidence between 1990 and 2008: lung (6,500), stomach (4,400), bladder (4,400), and cervical (1,400) cancers. Additional file 2 gives full details for the 21 cancers. The literature was consulted to identify possible causes for these reductions in incidence. Overwhelmingly, smoking prevention and cessation was cited as the reason for a reduction in lung cancers [30]. Falls in rates of stomach cancer are also thought to be linked to smoking along with declines in *Helicobacter pylori* and improvements in diet [29,31]. The picture is less clear, given changes in the ways these cancers are coded, but bladder cancer has been shown to be associated with smoking too [32], which may account for the decline in rates. The fall in cervical cancer can be largely attributed to the roll-out of cervical screening since the 1980s, which in addition to detecting cancers, is able to pick up pre-cancerous abnormalities and so reduce the incidence of cancer. This has led to a focus on reduction in smoking and on cervical screening.

In addition to cervical screening (which has been in its present form since 1988), there are currently two other national screening programmes in the UK aimed at early detection of cancers: breast cancer screening (introduced in 1988) and colorectal cancer screening (introduced in 2006). There is evidence that all three programmes have reduced mortality [33-35], and should be included in our list of priority interventions.

There have been substantial advances in cancer treatment in recent decades, which have led to valuable health gains. Surgical techniques remain a cornerstone of treatment, aided by ever-refined radiotherapy methods. The advent of new cytotoxic therapies, as well as hormonal and biological therapies, has greatly increased the available treatment options. Given the breadth of these treatments (and backed up by the number of treatments that expert opinion had identified) it was necessary to limit the focus of our estimation to a subset, which we expected to include most of the health gains likely to have been observed between 1991 and 2010. Data on changes in survival were used as a proxy for health gains. Data were compiled for cancer types on 1-year and 5-year survival rates from CRUK [36] and the Office for National Statistics (ONS) [37] (see Additional file 2). Rates were calculated as percentages for the period 1986 to 1990, and compared with those in 2005 to 2009 to calculate a change in the proportion of people surviving 1 and 5 years after diagnosis. This change in rate was then multiplied by the ‘mid-point’ incidence in 1999 to 2001 to estimate the additional number of people surviving. The same three cancer types (albeit in slightly different order) were found to have the highest number of additional people surviving for both 1 and 5 years; these were prostate, colorectal, and breast cancer. These three accounted for 73% of the estimated gains in 5-year survival. Using clinical guidelines published by the National Institute for Health and Care Excellence (NICE) a set of the main interventions for each of these three cancer types was identified. These interventions were all treatments, because, although there have been improvements in diagnostics and service configuration, it was assumed that the benefits derived from these should, in principle at least, be reflected in the number of people accessing treatment and in measures of treatment effectiveness.

### Identifying estimates of per-patient NMB for the set of cancer interventions

As a result of the approach outlined above, estimates of per patient cost and effects were then obtained from published studies for the following prioritised areas:

• Smoking prevention/cessation

• Screening programmes: cervical, breast, and bowel cancer.

• Treatment of: breast, colorectal and prostate cancer.

#### Smoking prevention/cessation

**Figure 1:**
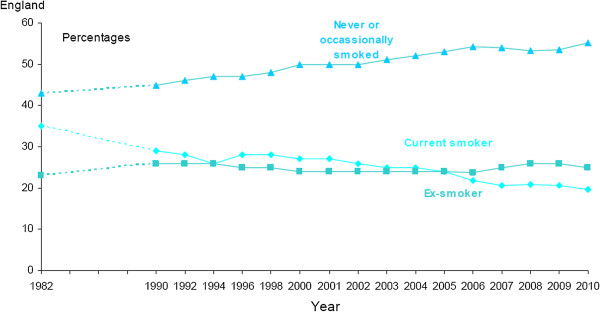
**Smoking behaviour in England, 1982 to 2010.** Source: General Lifestyle Survey 2010. The Office for National Statistics. Copyright © 2012, re-used with the permission of The Office for National Statistics.

The area where we adopted a very different approach to that which we had previously used for CVD was smoking. In that study, we restricted the analysis to the costs and benefits arising from NHS smoking cessation interventions. Cancer research has not only unequivocally shown the causal link between smoking (both active and passive) and both cancer and the risks of cancer (and other health problems) but also the effectiveness of various national interventions in reducing smoking rates. This cumulative evidence has contributed to a slow but steady change in smoking behaviour both through direct effects on individual behaviours and through the many non-NHS interventions in the UK (such as legislation and taxation) which have followed from, and been made possible by, this evidence, and have encouraged existing smokers to quit and discouraged others from taking up smoking, as summarised in Figure 1. Therefore, health gains from research include not only the benefit from getting smokers to quit (aided or not by the NHS), but also in preventing non-smokers from ever starting smoking. A recent modelling study for the UK DH Policy Research Programme provided estimates of lifetime life years gained and cost savings to the NHS of non-smokers and ex-smokers compared with smokers [38]. The model accounted for the mortality benefits from not smoking associated with lung cancer, myocardial infarction, stroke, and chronic obstructive pulmonary disease. In the absence of age-specific smoking rates, we used the estimates for men and women aged 35 years, and adjusted these to take account of the proportion of life years gained resulting from lung cancer reduction and also the adjusted life years gained by the population mean utility values for the relevant ages in order to estimate QALYs gained [39].

#### Screening programmes

To estimate the NMB of each of the three screening programmes, we identified the most appropriate economic evaluations that modelled the lifetime costs and effectiveness of offering the screening programmes as delivered in the UK. For both cervical and bowel cancer screening we used assessments that had informed relevant screening policy decisions [40,41]. In the case of cervical screening, we adjusted the figures presented as life years gained by an appropriate age/sex population utility values to give an estimate of QALYs [39]. For breast cancer, we used a recently published economic evaluation that had used a life-table model to assess the overall cost-effectiveness of the NHS screening programme which based its assessment of effectiveness on the findings of the Independent UK Panel on Breast Cancer Screening, and took account of the uncertainty of associated estimates of benefits, harms, and costs [33,42]. In all three cases, these models used take-up rates that were the same or very similar to those observed in the relevant screening programme during the period in question.

#### Treatment programmes

The full list of treatment interventions included in the estimation of health gains for each cancer site are shown in Additional file 3. These were determined based on NICE Clinical Guidelines (CG131 for colorectal cancer [43], CG80 and CG81 for breast cancer [44,45] and CG58 for prostate cancer [46]) and cross-checked to ensure that relevant interventions identified by experts were included. Patient sub-groups were recognised where distinction in treatments was made, or where likely differences in cost and benefits existed. In breast cancer, for instance, this distinction was made for node-positive cancers, oestrogen receptor-positive cancers, HER-2-expressing cancers, and pre/post-menopausal incidence of cancers, and between early-stage and late-stage cancer. Historical comparators for each intervention identified from the contemporary guidelines were then identified back to 1991.

For each of the treatment options considered, published economic evaluations were used to estimate per patient costs and benefits (measured as QALYs). Searches were conducted using the NHS Economic Evaluation Database and MEDLINE to identify economic evaluations of prostate, breast, and colorectal cancer interventions. UK-specific estimates were preferred, but international evidence was used where no appropriate UK estimates were available. Where they were available, NICE technology appraisals and National Institute of Health (NIHR) Health Technology Assessments were used as the most relevant sources (see Additional file 3). Where exceptionally non-UK cost-effectiveness data had to be used, costs were converted using purchasing power parity exchange rates.

### Constructing a time series (1991 to 2010) of usage of cancer interventions

To estimate total NMB for the period, per-patient QALY gains and net costs for each intervention were multiplied by the total number of new patients receiving each intervention in each year. We used the following methods to estimate the time series of usage for the selected interventions.

For smoking reduction/cessation we used figures derived from the data on the proportions of smokers, ex-smokers and non-smokers for England for each of the years to estimate the net change per year in QALYs gained and NHS savings achieved, and related these to population data for the UK as a whole [47].

For cervical and breast screening programmes, we used figures for the relevant size of the UK age group in each year to whom screening was first offered (age 25 for cervical and age 50 for breast). For bowel screening we used the numbers first offered screening as the programme began to be rolled out.

To estimate the numbers of people receiving each treatment intervention over time two primary sources were used. For surgical procedures (for example, colorectal excision, liver resection and ablation, prostatectomy, orchiectomy, mastectomy and lumpectomy) Hospital Episodes Statistics [48] were utilised. To estimate the numbers of people receiving drug interventions, data on Net Ingredient Cost (NIC) of drugs to the NHS were used. These data were gathered from Health and Social Care Information Centre (HSCIC) data publications [49], which give details of the total cost of a particular drug prescribed in primary care (for the Prescription Cost Analysis) and secondary care (Hospital Prescriptions Audit Index) in each year. For some drugs, this information was not available for the whole of the time period, in which case assumptions were made on the basis of launch year and the most recent available time point. If the launch year occurred during the period 1991 to 2010, a linear interpolation with launch year at £0 NIC was performed. For drugs that were not launched during the period, a last value carried back approach was adopted, using the most recent year of historical data. From the NIC, the cost and length of a typical regimen (as estimated by NICE costing templates where possible) were used to calculate the number of complete treatments delivered and hence the number of people receiving a particular drug in any given year. This was then proportioned across the indications of a drug and particular patient group (for example, early and late cancers, or multiple cancers).

For some older drug interventions, NIC data were not publicly available for any of the years of interest. In these instances, NICE estimates of the proportion of patients likely to receive interventions (based on guidance costing templates) were combined with data on incidence to estimate usage numbers.

For radiotherapy, there was a paucity of data on usage. Data from the National Clinical Analysis and Specialised Applications Team (NATCANSAT) were available for 2009/10, giving the number of episodes of radiotherapy.^e^ It was estimated that 70% of these episodes would be for primary treatment of a cancer. The number of primary radiotherapy episodes was estimated as a proportion of the incidence of each cancer in 2009/10. This proportion was applied historically to incidence in order to estimate radiotherapy treatment.

The component figures of numbers of people receiving treatment interventions were all derived from data for England. To produce a UK estimate (needed because the research spend data is for the UK) figures were adjusted by a factor reflecting England’s proportion of the adult UK population. The screening was based directly on relevant UK population data, and for smoking behaviour the time series data were for England, but have been applied to the UK population. All cost estimates were adjusted to 2011/12 prices using the Hospital and Community Health Services Pay and Prices Index [50].

For the calculation of NMB, we used for the base case an opportunity cost value of a QALY as used by NICE in its decision-making [51,52]. This value reflects an estimate of the opportunity cost in terms of QALYs forgone elsewhere in the health service within its fixed budget. Given that public spending on health research can justifiably be seen as a decision to spend on research rather than directly on current healthcare, this opportunity cost value is appropriate to the public decision regarding research funding. In this study, as previously for CVD, we characterised NICE’s threshold range as equivalent to an average of £25,000 per QALY, but considered a broader range of values in the sensitivity analysis, including a value of £70,000, which would be broadly consistent with the commonly proposed QALY threshold of 3 times GDP per capita [53].

### Analysis of UK clinical guidelines to estimate elapsed time and rate of attribution

In the 2008 report on CVD research, the references cited in a sample of clinical guidelines were analysed to inform the estimate of the elapsed time between research spend and net health gain, and the proportion of net health gain that could be attributed to UK research [3]. In the current study on cancer research, we replicated this approach.

In total, 31 national clinical guidelines, which provided a broad representation of cancer practice in the UK, were identified. Twelve were published by NICE and a further twelve by the Scottish Intercollegiate Guideline Network (SIGN). The remaining seven guidelines were published by either the Royal Colleges or the National Cancer Screening Programme. The reference sections of these guidelines were reviewed: five had no reference list (four published by NICE, one by the National Screening Programme) while one screening guideline had no references to peer-review journals (that is, it referenced only policy and practice documents). These six guidelines were excluded from our sample. We then used a bespoke computer programme to extract references from the electronic PDF version of each guideline; in three cases the automated reference extraction failed (because papers were not referenced in a recognised format), leaving us with a sample of 22 national guidelines.

Of the 5,627 references cited in the 22 guidelines, 4,416 references (78%) were automatically extracted, excluding duplicate references within a guideline (see Additional file 4 for breakdown by guideline). Nine of these references had no date information and were excluded from the analysis of elapsed time, leaving a total of 4,407 references. The age of a paper cited in a clinical guideline has been termed the ‘knowledge cycle time’ [54], which is the average difference between the publication date of the clinical guideline and the publication date of the cited papers on the guideline. The knowledge cycle time was calculated for the 22 identified guidelines, and used to inform the estimated elapsed time.

To estimate the rate of attribution to the UK, the 4,416 extracted and de-duplicated references were provided to the Centre for Science and Technology Studies (CWTS) to be matched to their bibliometric database (which is derived from the Web of Science).^f^ Of the 4,416 extracted references, CWTS was able to match 4,051 (92%), which formed the dataset to estimate the degree of attribution based on the address field in the cited papers. These addresses were used as a proxy for the location in which the research was conducted, and so it was possible to estimate the proportion of the cited research that was conducted in the UK. The non-matched references included non-serial outputs such as books, journals that are not indexed on the Web of Science, and incorrect references.

### Estimation of the rate of return

Using these four key sources of data, we could then attribute a proportion of the estimated total annual NMB of the cancer health gain as being due to UK research, and relate an equal number of years of investment to years of NMB, ‘lagged’ by an estimate of the average lag between research and benefit. The return was expressed as an IRR, which is effectively the discount rate that would yield a zero net present value. The IRR is convenient in enabling a comparison to be made between non-competing investments of different sizes (as well as providing a direct comparison with our previous study). We recognise the many and various layers of estimates involved. In other circumstances, it might be feasible to express the uncertainty as ranges for each parameter in our overall estimate and undertake a formal probabilistic sensitivity analysis (PSA). However, given the nature of the evidence from multiple sources for the numerous parameters and the necessary judgments involved in drawing together and interpreting the evidence, a comprehensive PSA quantitatively characterising all the uncertainty was not feasible here, and indeed would be liable to suggest a spurious precision. Instead we provide a series of one-way and scenario sensitivity analyses to illustrate the effects of specific variables on the IRR.

## Results

### Public and charitable funding of UK cancer-related research, 1970 to 2011

**Figure 2:**
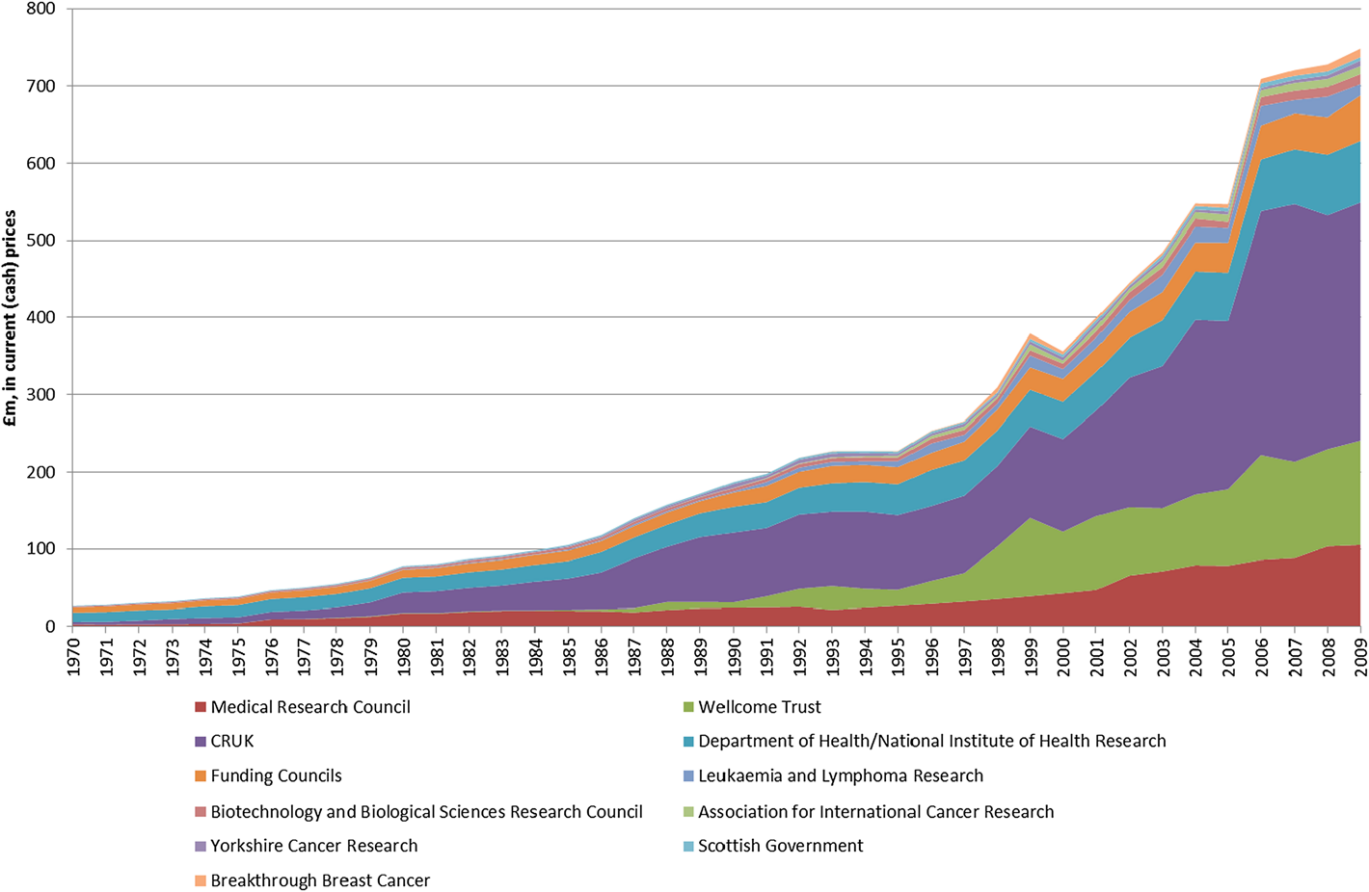
Total estimated public and charitable spend on cancer research by source of funding, 1970 to 2009, in current (cash) prices.

**Figure 3:**
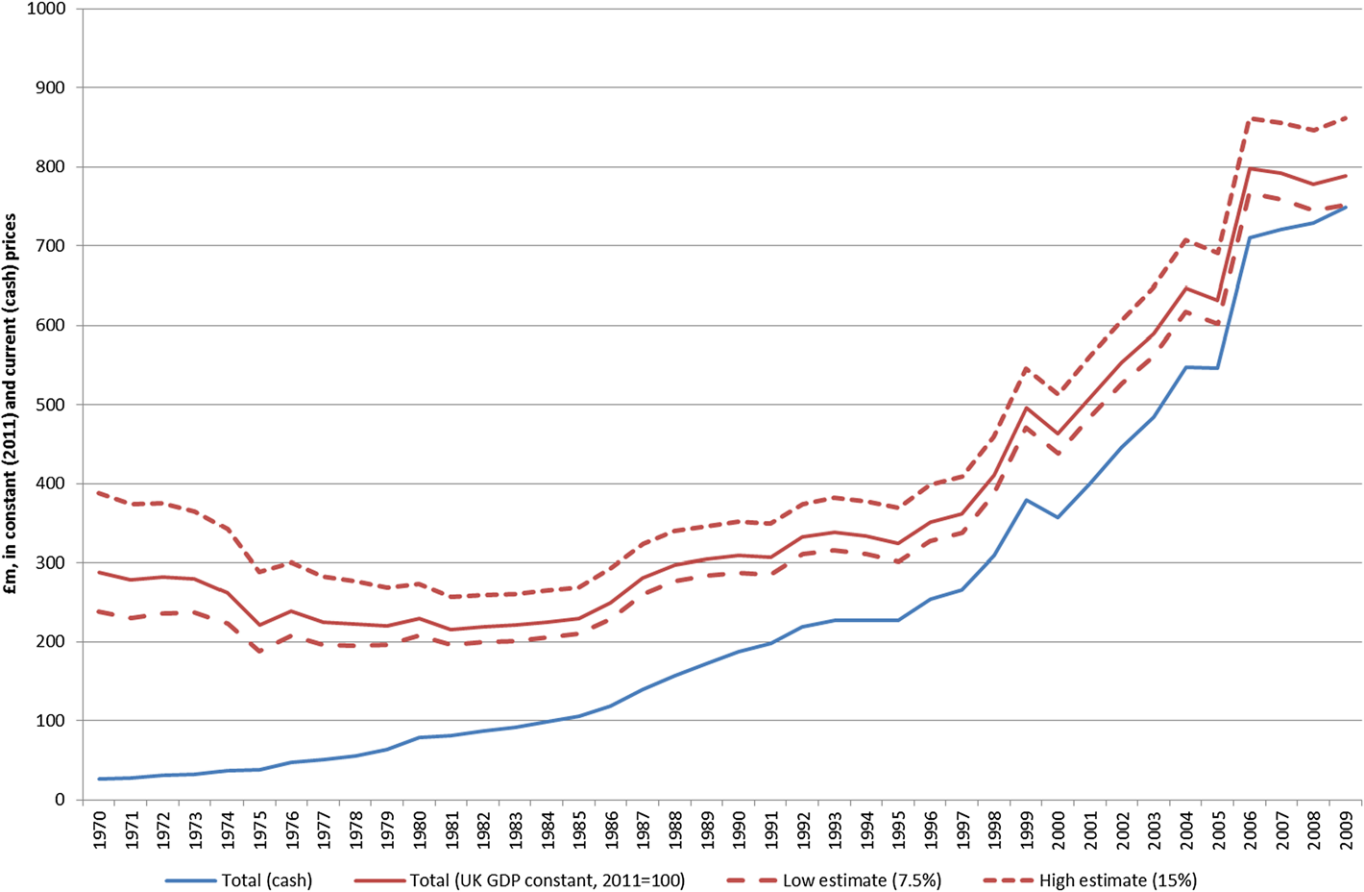
Public and charitable funding of cancer research 1970 to 2009, at constant (2011) prices with low and high estimates, and in current (cash) prices.

Additional file 1 provides our estimated expenditure by year by organisation for the 40-year period, 1970 to 2009, with a summary of cash expenditure provided in Figure 2. Figure 3 illustrates estimated public and charitable expenditure on cancer-related research from 1970 to 2009 in cash and constant 2011/12 prices (the latter for our best estimate). About £15 billion (in 2011/12 prices^g^) of cancer-related research funding was invested during this period. The data presented in Figure 3 are derived from a number of different sources and include various assumptions and estimations. For this reason, we also provided a ‘high’ and ‘low’ scenario for total cancer-related research expenditure with a range of £14 to £17 billion. In Figure 3, we also present total public and charitable spending on cancer-related research in cash terms. This emphasises that in real terms (in 2011 prices; the red line) spending fell between 1970 and 1979, then stagnated until 1986, and thereafter increased threefold, from £250 to £850 million, by the end of the time series in 2009.

### Net monetary benefit

Table 2 shows the contributions to our total estimates of lifetime QALYs gained from the seven areas we addressed, classified by the year in which the intervention was delivered (or in the case of screening, the year in which those targeted entered the screening programme). Reduction in smoking accounted for 51% of the QALYs gained from the seven areas we prioritised. The other two large sources of QALYs gained were from cervical screening (21%) and breast cancer treatments (19%). The other areas we examined were small contributors by comparison.

**Table 2:** Contributions of the seven areas to the total estimates of lifetime QALYs gained by year: 1991 to 2010

	**QALYs (thousands)**							
**Year**	**Treatment**			**Screening**			**Smoking reduction**	**Total**
	**Prostate cancer**	**Breast cancer**	**Colorectal cancer**	**Cervical cancer**	**Bowel cancer**	**Breast cancer**		
1991	8	46	6	71	–	2	144	277
1992	9	48	6	70	–	2	144	279
1993	10	46	6	68	–	2	145	276
1994	11	48	6	67	–	2	145	279
1995	10	45	6	65	–	2	145	273
1996	11	46	6	66	–	2	146	276
1997	11	46	6	63	–	3	146	274
1998	11	50	6	59	–	2	147	276
1999	13	53	7	56	–	2	147	279
2000	15	53	7	55	–	2	148	281
2001	17	55	7	54	–	2	149	285
2002	20	56	8	53	–	2	150	290
2003	22	59	8	53	–	2	151	295
2004	24	61	9	56	–	2	152	305
2005	21	62	10	60	–	2	154	309
2006	22	62	12	61	2	2	155	316
2007	23	65	13	60	5	2	157	324
2008	27	68	14	60	7	2	158	337
2009	26	71	15	63	9	2	159	345
2010	25	74	15	65	12	3	161	354
Total	339	1112	173	1225	35	43	3003	5930

*Abbreviation:QALY* quality adjusted life year.

Table 3 shows the lifetime net costs to the NHS for each of these areas over the 20-year period. The key points to note here are the high proportion of total net costs accounted for by breast cancer and prostate cancer treatments. Smoking reduction on the other hand reduces net NHS costs, as does colorectal screening, although the latter’s introduction late in the period covered means its absolute contribution to reducing overall costs is small.

**Table 3:** Contributions of the seven areas to the estimates of lifetime costs to the NHS of services delivered by year: 1991 to 2010

	**Costs (GB£ million)**							
**Year**	**Treatment**			**Screening**			**Smoking reduction**	**Total**
	**Prostate cancer**	**Breast cancer**	**Colorectal cancer**	**Cervical cancer**	**Bowel cancer**	**Breast cancer**		
1991	199	665	181	41	–	34	-277	844
1992	220	687	190	40	–	37	-277	897
1993	241	658	185	39	–	40	-278	887
1994	272	684	185	39	–	42	-278	944
1995	252	646	175	37	–	42	-279	874
1996	278	660	182	38	–	43	-280	921
1997	269	684	181	36	–	53	-281	943
1998	283	720	187	34	–	50	-282	993
1999	336	753	214	32	–	47	-283	1098
2000	391	746	211	32	–	45	-285	1140
2001	456	755	206	31	–	43	-287	1204
2002	519	764	202	30	–	43	-282	1276
2003	571	794	178	30	–	44	-255	1361
2004	614	817	179	32	–	44	-254	1432
2005	545	850	182	34	–	44	-252	1403
2006	572	851	188	35	-5	45	-258	1428
2007	596	881	177	35	-10	46	-252	1473
2008	613	919	181	35	-15	48	-242	1538
2009	606	950	185	36	-20	48	-237	1569
2010	569	986	186	38	-25	56	-240	1569
Total	8403	15469	3755	704	-75	894	-5358	23793

*Abbreviation:NHS* National Health Service.

Table 4 summarises the NMB when the QALYs have been valued at £25,000 and the net costs to the NHS of the intervention and its long-term sequelae have been deducted. It shows how the total NMB (when measured in constant prices) from the research-based interventions that we have assessed has been steadily increasing, with an overall increase of 28% over the 20-year period. Over the whole period, smoking reduction (providing for both QALYs and NHS cost savings) accounted for 65% of NMB, followed by cervical screening (24%) and breast cancer treatments (10%). All seven areas we studied showed a positive NMB when QALYs were valued at £25,000. However, at a QALY value of £20,000, prostate and colorectal treatments and breast cancer screening all showed a negative NMB (that is, their net costs exceeded the valuation of the benefits they provide).

**Table 4:** Contribution of the seven areas to the estimates of net monetary benefit by year: 1991 to 2010 (QALY value of £25,000)

	**Net monetary benefit (£ million)**							
**Year**	**Treatment**			**Screening**			**Smoking reduction**	**Total**
	**Prostate cancer**	**Breast cancer**	**Colorectal cancer**	**Cervical cancer**	**Bowel cancer**	**Breast cancer**		
1991	8	490	-33	1729	–	7	3885	6085
1992	8	506	-35	1699	–	7	3889	6075
1993	9	485	-33	1659	–	8	3890	6018
1994	10	504	-32	1646	–	8	3894	6030
1995	9	475	-28	1592	–	8	3906	5963
1996	-3	483	-28	1609	–	9	3920	5989
1997	-3	458	-28	1546	–	11	3933	5916
1998	-3	531	-29	1447	–	10	3947	5902
1999	-10	566	-40	1374	–	9	3966	5866
2000	-15	579	-36	1350	–	9	3989	5876
2001	-25	630	-30	1310	–	9	4018	5910
2002	-8	647	-5	1283	–	9	4037	5963
2003	-14	677	24	1294	–	9	4035	6025
2004	-7	705	49	1376	–	9	4064	6195
2005	-15	694	67	1460	–	9	4099	6314
2006	-16	697	106	1484	64	9	4138	6481
2007	-23	736	149	1469	128	9	4167	6635
2008	51	786	171	1474	192	10	4192	6876
2009	46	816	178	1528	256	10	4216	7050
2010	66	854	183	1596	320	11	4253	7282
Total	65	12318	566	29927	960	179	80437	124452

*Abbreviation:QALY* quality adjusted life year.

### Estimating the elapsed time

**Figure 4:**
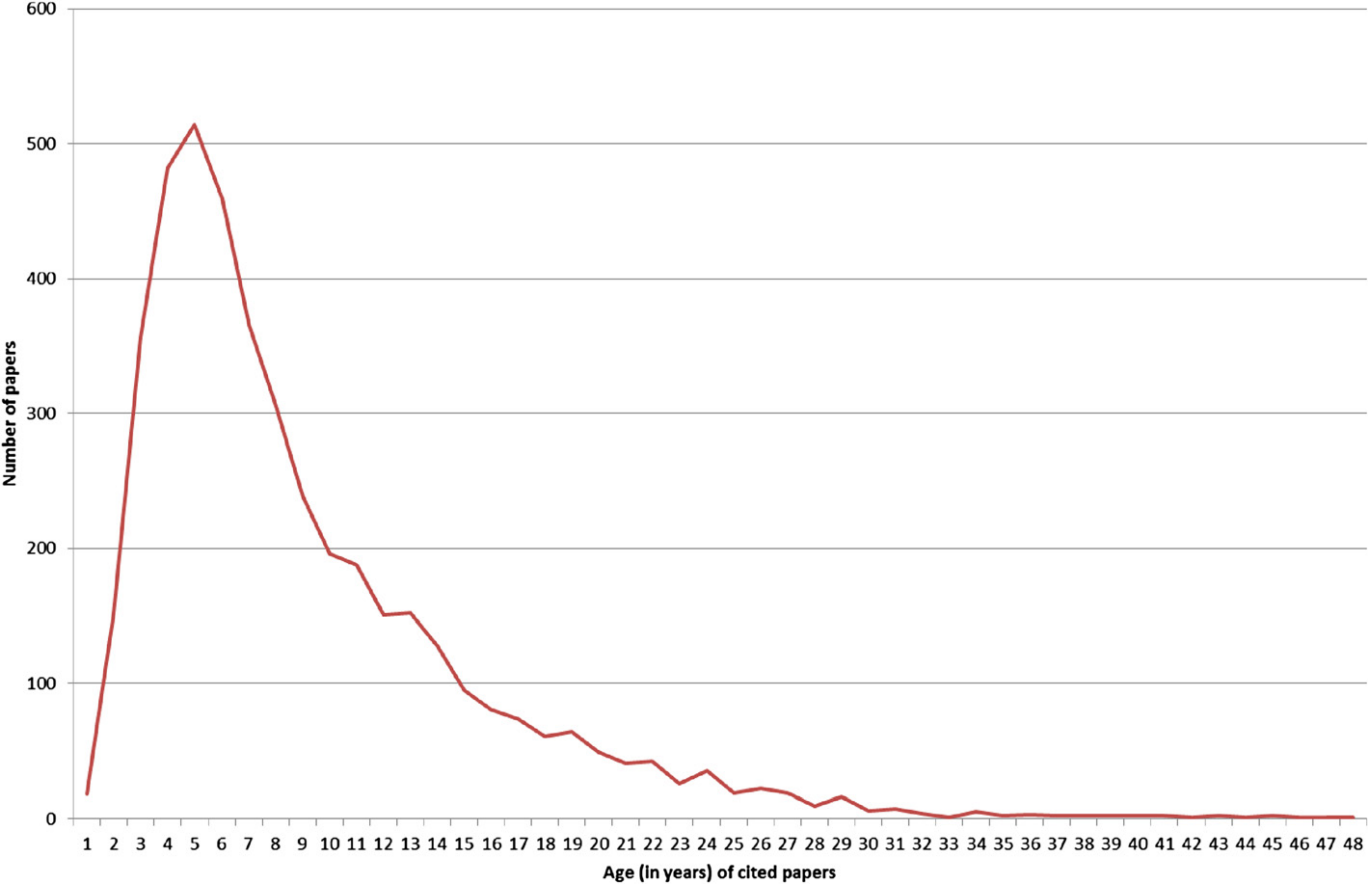
Age of papers cited on cancer clinical guidelines.

The estimate of the elapsed time used in the study was based primarily on the analysis of cited references on clinical guidelines (that is, knowledge cycle time). As illustrated in Figure 4, the mean age of the 4,407 cited papers on the 22 guidelines was 8 years, ranging from 0 to 88 years (the median age was 6 years, with an interquartile range of 3 to 10 years). To produce an estimate of elapsed time between spending on research and health gain as required for this study, it was necessary to add on to this value the estimates for the period between the awarding of funding and publication, and the period between recommendation and use. Using the same approach adopted in the 2008 report, we estimated these two periods to total approximately 7 years, giving a best estimated elapsed time between spending on research and health gain of 15 years, with 10 and 20 years arbitrarily selected as lower and higher estimates for sensitivity analyses.

### Estimating the amount of health gains that can be attributed to UK research

The estimate of the proportion of the health gain that can be attributed to UK research used in the study was based primarily on the analysis of cited references on clinical guidelines. A total of 4,051 publications were analysed to estimate the proportion of the research that could be attributed to the UK. The overall percentage across all guidelines was 17%, but as shown in Additional file 4, this differed between specific guidelines.

### Estimating the IRR from cancer-related research

Our estimates of the NMB produced by year (summarised in Table 4) were then related to the estimated public and charitable spend by year on cancer-related research (summarised in Figure 3) and expressed as an IRR. Calculation of the IRR incorporates our best estimates of the elapsed time of 15 years (low and high estimates of 10 and 20 years) and of the proportion of the NMB that could be attributable to UK research (best estimate 17%: low and high range estimates of 10 and 25%). Thus in our base-case calculation we related 17% of the annual NMB (for each of the 20 years 1991 to 2010) to 20 years of the research investment that had occurred 15 years earlier (that is, for the years 1976 to 1995; in other words, a subset of the 1971 to 2009 series collated). This produced a base-case estimate of the IRR of 10.1%.

As is evident from the methods used, there is inevitably considerable uncertainty around the values of all our estimates. Table 5 presents a series of one-way sensitivity analyses to illustrate the effects of some of the main areas of uncertainty, and all changes have predictable effects. For NMB, the greatest uncertainty in our calculations probably relates to the magnitude of the benefits from smoking, given the indirect nature of the estimate. Reducing (or increasing) the NMB from smoking by 25% produced an IRR of 8.7% (or 11.2%); for illustration, the (unrealistic) extreme of removing entirely the benefits from smoking from our estimates produced an IRR of 2.4%. The IRR increased as our estimates of research funding were reduced, and the proportion of benefits attributable to UK research increased. It was found to be particularly sensitive to a reduction in the elapsed time. Although taken individually, all of the alternative values we have explored in this sensitivity analysis showed a reasonable rate of return, in combination they could of course have produced a wider range of estimates for the IRR.

**Table 5:** IRR: one-way sensitivity analyses

**Analysis**	**IRR, %**
Base case^a^	10.1
Research funding estimate	
Low	10.8
High	8.7
‘Value’ of a QALY, GB£	
20,000	8.0
30,000	11.7
50,000	16.1
70,000	18.9
Elapsed time, years	
10	14.6
20	7.4
Attribution to UK research, %	
10	6.1
25	13.0
Effect of smoking cessation	
Decrease NMB by 25%	8.7
Increase NMB by 25%	11.2
Omitting benefit of smoking reduction	2.4

*Abbreviations:IRR* internal rate of return, *NMB* net monetary benefits, *QALY* quality adjusted life year.

^a^Best estimate for research funding and net monetary benefit, QALY value of £25,000, elapsed time of 15 years, and attribution to UK research of 17%).

## Discussion

Taking into account the necessary assumptions made in our approach, the base-case IRR for the NMB from the health gain from cancer research of approximately 10% is remarkably similar to that derived for CVD research, where the IRR derived from the health gain was 9%. These benefits alone provide a return considerably greater than the UK government’s minimum threshold of 3.5% for investments, thus suggesting that investment in cancer research is worthwhile. Moreover, given that CVD, cancer, and mental health account for about 45% of the total burden of disease in the UK [55], we might with increasing confidence extrapolate this order of rates of return to the whole of the public and charitable investments in biomedical and health research in the UK. The important caveat to that statement is that the two of the clinical areas that we have analysed in most detail – cancer and CVD –have both benefitted significantly from the changes in smoking over the period analysed.

However, it should be remembered that in our previous study, the rate of return from NMBs of the health gain was less than a third of the rate of return (30%) that we suggested might relate to the broader GDP gains. If we accept that estimate of returns from GDP, then again the overall returns from cancer research would be in the order of 40%. However, as we noted then, although this estimate was based on the best available information, it was generated from a small empirical literature, much of it US-centred and only a proportion specific to medical research. From the papers reviewed, a rate of return between 20% and 67% was identified and we took 30% as our ‘best estimate’. The current study did not revisit this aspect of the return to investment. As discussed below, we recommend that future research should aim to update and improve on these estimates.

### What this paper contributes

In this study, our main methodological contribution was to further validate the bottom-up approach we developed in the original *Medical Research: What’s it Worth?* study [3]. This new application strengthens our argument that the bottom-up approach represents a significant improvement on earlier attempts to estimate economic returns from research, as it attempts to directly attribute health gains (as measured by QALYs) to research-derived interventions. The alternative ‘top-down’ approaches face the fundamental problem of starting with changes in mortality or morbidity over time, and attributing an estimated proportion of these changes to biomedical and health research. In addition, and in line with our previous work, we have taken into account the costs of delivering the health gain and the elapsed time between research investment and health gain, which earlier studies had largely failed to do.

### Key assumptions and caveats

Despite validating and further developing the approach, there are still a number of key assumptions and caveats in our estimate of the economic returns from cancer-related research. Given this, we would be the first to acknowledge that the bottom-up approach by necessity relies on these assumptions, and that our findings do need to be treated with appropriate caution. We document these assumptions in the interests of transparency, and to stimulate further research. The key assumptions are as follows.

• **Our base-case value of a QALY is £25,000.** Obviously, and as demonstrated by our sensitivity analysis, the IRR is sensitive to the assumed value of the health gain measured as QALYs. Our base-case assumption is consistent with our analysis of the returns to CVD research, and reflects the mid-point in the range of values (of £20,000 to £30,000) cited as the normal criteria for acceptance of interventions by NICE [51]. More recently, NICE has increased this threshold, up to around £50,000, for certain treatments that provide end-of-life benefits, particularly late-stage cancer treatments [52]. At the same time it has seemed to re-emphasise that the £20,000 threshold should apply unless there are special circumstances. Although this leaves uncertainty about the most appropriate value here (as reflected in our sensitivity analysis), conceptually the argument remains that this ‘opportunity cost’ value of a QALY should apply to an assessment of research in that investing in health-related research can be seen as an alternative to spending the money directly on current health care. We note, however, that other studies in the US and Australia have used much higher values, reflecting individual willingness to pay for health gains, and we have illustrated in a sensitivity analysis the effect of using a value of the order of three times GDP per capita [53].

• **The total NMB for interventions not covered is assumed to be zero.** Our IRR calculation assumes that all other cancer treatment developments/interventions that we have not specifically included have, in aggregate, no effect on the NMB, because for these, the monetised value of the health benefit is equal to the cost of delivering the benefit. In reality, there may be some areas that we have not covered for which the NMB is negative because of the high cost of treatment and low incremental health gain. Conversely, there are may be other areas that generate a significant number of QALYs at a relatively low cost. We are not in a position to know whether the net effect of the interventions we did not examine is positive, negative, or zero.

• **The total net flow of knowledge between disciplines is zero.** We have assumed that the flow of knowledge is the same into and out of different research fields, and from each research field into the cognate treatment areas. However, we know that research is unpredictable and diffuse, and there may be research disciplines that contribute more than they gain from other areas. One could argue that some of the reduction in mortality from diseases other than cancer that arises as a result of the reduction in smoking (e.g. CVD) which we have excluded, should in fact be included as having been achieved as an additional advantage arising from the evidence of the effect of smoking on lung cancer.

• **All health gain from treatments is captured in the estimates of the health gain from specific interventions.** We have assumed that in principle the health gain from improved service configuration and all other supportive service changes (including diagnostics and imaging) should be captured in the estimates of the gains from specific interventions. In practice, our estimates of QALY gains are mainly derived from UK-relevant health technology assessments that are extrapolated from trial data, which may provide an imperfect estimate of the gain when the interventions are used in routine NHS practice.

• **The definitions of the cancer-related research used by the research funders captures basic research that may have contributed to developments in this area.** This is clearly the case for the cancer-specific funders such as CRUK, as we included all the research they funded. For MRC funding, we relied on the funder classification which, as discussed in Additional file 1, was broad and thus should include basic research. For the Wellcome Trust, which accounts for around 10% of total cancer funding, we had to rely on search terms. We scanned the list of grant titles selected through this search strategy, and this list suggests that fundamental research is being included, although we cannot guarantee that it all is in fact included. For the remaining two funders – the Funding Councils and the DH/NHS – this would not be an issue, as their time series were derived through an estimate of cancer research activity.

• **The knowledge cycle time and attribution rate were largely determined through bibliometric analysis of clinical guidelines.** As part of this study, and reported separately, we undertook a series of case studies that qualitatively explored how research translates into health benefit [24]. This work demonstrates the complexity of biomedical and health innovation, especially when trying to measure the time it takes for research to develop into health benefits. Although the bibliometric approach provides us with an empirical estimate of both the elapsed time and the rate by which we can attribute UK research to UK health gain, it inevitably is a gross simplification of a complex process.

• **We have made various assumptions about the baseline treatment against which we were looking in research-based developments.** For example, in estimating the net health gain from breast cancer treatments, we did not include benefits from standard mastectomy but just estimated the benefits from subsequent developments.

• **There is a risk that we may have double-counted the NMB for individuals who are treated as a result of screening.** Conceptually, the benefits of screening include the downstream NMB of treatments that result from the screening. However, a number of issues minimise the likelihood of our double counting. First, we did not include (in the treatment calculation) all the benefits of treating an individual disease (for example, breast cancer) but only the additional benefits of improved (research-based) treatments, so any additional people who get ‘basic treatment’ as a result of screening were counted only as an advantage to screening. Second, the benefits and the future treatment costs of a woman entering a screening programme (which is when we estimated the future QALYs and present value of associated net costs) occur in a future year, often many years ahead, so in taking a 20 year period, there is limited scope for counting both. If we had perfect data and were looking at all treatment benefits over a much longer period, we could in principle look only at the benefits of treatments that would encapsulate all the QALY benefits of screening.

In acknowledging these assumptions, we should make the important point that an underlying principle we adopted throughout this study and our previous work on CVD was to err on the side of caution: that is, to make assumptions that would lead, other things being equal, to a lower rate of return. However, compared with our earlier study of CVD, we are less confident that we have always managed to adhere to the principle of conservatism. For example, as discussed above, there is an implicit assumption in ascribing the IRR to the whole of cancer that everything we have not specifically included has, in aggregate, no effect on the NMB (the value of the health gain is equal to the costs of delivering it). In reality, the aggregate effect of what we have not considered could be positive, negative, or zero. Another issue is that in the CVD study, conservatism often came from adopting the lower of two (or more) published estimates for specific parameters, but for cancer interventions, we rarely had a choice of relevant data estimates, as discussed in more depth below.

In addition to these specific assumptions, there are a number of other broader issues that add to the uncertainty of our estimates and need to be highlighted.

• **We have evidence of linkage between research and health gains but no formal evidence of causality.** Our analysis relied on the reasonable assumption that these health benefits would not have occurred without the evidence from medical research, and we have illustrated the often complex nature of those linkages in case studies [24]. At one level we have addressed this issue of causality by our bottom-up approach, adding together the benefits demonstrated through clinical trials of new interventions. For these, causality from worldwide medical research is all but a truism. However, even for these, we had to assume that a proportion of the benefit (based on the UK contribution to publications cited in guidelines) arose from UK research. It is possible that some or even all of these interventions might have come into use in the UK even if there had been no UK cancer research, but it is improbable that the same level and timing of benefits would have arisen. Causality could be argued to be less direct for the benefits of the reduction in smoking, which made the largest contribution to the total NMB. It is possible, but implausible, that changes in smoking behaviour might have arisen in the absence of any evidence of the health effects. Certainly, our case studies [24] show that there was an extended lag between the initial evidence of harms to smokers and changes in behaviour, and the UK government probably needed the cumulative evidence that has emerged over several decades, and in particular the evidence of the harms of environmental tobacco smoke, to make the legislative changes in the face of very considerable resistance. There are also additional uncertainties around the magnitudes of NMB from smoking. Of the total £124 billion total NMB, £80 billion (or 65%) arose from reductions in smoking, and the numbers for the increased proportion of the population who were non-smokers or ex-smokers is based on self-reported survey data. In the sensitivity analysis (Table 5), if the NMB from smoking reduction was decreased or increased by (an arbitrary) 25%, the IRR would reduce to 8.7% or increase to 11.2% respectively. Omitting the benefits from smoking reduction entirely reduces the IRR to 2.4%. However, it should be stressed that we estimated only the mortality effects on lung cancer and excluded effects on other cancers (and other disease areas) from smoking, all of which would mean we probably underestimated the impact of smoking reduction. However, taking a perspective of NHS costs only, we have not included costs to other parts of the economy from the various measures to reduce smoking [56].

• **Variable quality of data on the effectiveness of screening.** The three national screening programmes are important elements in our estimates. The clinical and cost-effectiveness evidence for bowel cancer screening is high-quality and trial-based, but the evidence for cervical screening, and even more so for breast cancer screening, is less robust. The recent review [33] of the clinical evidence has provided some clarity to the contentious issue of the net benefits of breast screening, and underpins the relatively simple economic model that we used as the basis of our estimate of NMB, but there is considerable uncertainty around these estimates.

• **There is a lack of robust clinical effectiveness and cost-effectiveness data for some interventions, especially for longstanding treatments.** This was a general problem with well-established surgical techniques (for example, total mesorectal excision, for which no cost-effectiveness evidence could be found) and similarly for some of the hormonal therapies (for example, tamoxifen and goserelin).

• **There are a large number of areas of cancer that we did not consider in our analysis.** Our analysis was based on a prioritised list of cancer types generated from both expert opinion and epidemiological data. By necessity, this meant we did not look at a number of areas (and as noted above, assumed the NMB arising from these areas to be zero).

• Elapsed time was an important variable in determining the IRR, but one that is conceptually difficult to measure [24]. We wanted to measure the time between research investment and health gain, but neither of these events occurs at one defined point. Research investment may occur over a period, although in many cases, given a typical pattern of investment starting with pilot trials, and building to larger-scale studies and finally randomised controlled trials, the bulk of the research investment may come late in the overall investment period. The point at which the bulk of the health gain occurs is even more difficult to define, and will depend on a range of factors, such as the type of intervention and the way in which it is implemented. The issue of time lags was identified in the original 2008 report, which suggested that further research is needed.

Given these various issues and the nature of the exercise, which relies on data and estimates from a wide variety of sources, it is not possible to characterise in any formal way the overall uncertainty in our estimates. The sensitivity analysis illustrates the effect on the IRR of alternative values for some of the key parameters, and shows that the broad order of magnitude of the IRR is relatively insensitive to fairly substantial degrees of uncertainty on specific elements of the analysis of what has happened in the past. Moreover, even without this uncertainty, we need to interpret our analysis of what has happened in the past with caution, as follows.

• **Past performance is not an indicator of future performance.** The IRR is based on past performance, and cannot be a guarantee of future returns, particularly for increased levels of research spending. This means that research advocates need to use the estimates provided in this paper very cautiously if wishing to extrapolate them as indicators of likely future returns from research expenditure. Given the near doubling in cancer-related research funding since the turn of the century (Figure 3), there will need to be a similar increase in NMB in the coming decade to maintain the current returns. It is worth noting that the NMB of bowel screening is not fully reflected in the IRR because this screening is of recent introduction, so there is additional benefit that will be realised in the future. Likewise, pharmaceutical interventions are typically priced to maximise the value of the benefit at time of introduction, so the NMB is close to zero. During the coming decade, some of the expensive drugs will come off patent and may be available more cheaply, thus contributing to an increase in the NMB; however, other new and expensive ‘on patent’ drugs may well be used in preference.

• **We estimated average returns from cancer research, not the marginal returns.** From this analysis, we are not able to say whether the rate of return would have been different if research spending had been higher or lower, and whether at the margin the returns to research investment are increasing or diminishing.

• **The analysis should not be used to make comparative assessments about the value of research into particular interventions/cancers.** Our approach examined a portfolio of interventions/cancer types and we would caution that the detailed data may not be sufficiently robust to make comparisons between interventions within specific cancers.

### Future research requirements

Based on the key assumptions, uncertainties and caveats described above, further research is needed in the following areas.

• **A deeper understanding of the international flows of knowledge.** In our model, we estimated the extent to which UK research influences UK practice, using citations on clinical guidelines, and this figure was used in estimating the IRR. However, there is a need for a more nuanced understanding of these knowledge flows and their impact on international health gains; for example, UK research is contributing to health gains beyond the UK. As a result, our current figure underestimates the global value of UK R&D. A study that aimed to measure the health gains, net of healthcare costs, in the rest of the world as a result of UK medical research would address this. At a European level, it would also be interesting to explore how the investments of different European countries in biomedical and health research leads to health gains in other European countries, thereby reinforcing the notion of European solidarity.

• **An improved estimate of spillover effects for UK biomedical and health research.** Public and charitable biomedical and health research expenditure not only leads to health gains, but also makes an important contribution to the national economy. Much of the evidence base for estimating a spillover effect of 30% comes from studies undertaken in the 1960s and 1970s, and/or relates specifically to agriculture research. More recent analyses for medical research are largely based on US data. Furthermore, in this study, we also assumed that the spillovers are independent of disease area but we have no empirical evidence to support whether that assumption is justified or not. Future research should aim to provide empirical estimates of the effects of biomedical and health research for the UK economy, ideally at a disease-specific level.

• **Examine another disease area or time period in which smoking reduction is likely to have a minimal impact.** As illustrated in Table 5, the IRR for cancer research is very dependent on the effect of smoking reduction. It would be valuable to undertake an investigation in another clinical area in which smoking is not important to see whether similar rates of return are found.

## Conclusion

It is challenging to move beyond the identification of the benefits from specific examples of research funding and attempt to meet the increasing demands for accountability by systematically measuring returns to the investment of a whole body of medical research. In this paper, we have estimated the economic benefit of public and charitable funding of cancer-related research, and further validated the methodological approach that we originally used in assessing the returns from CVD research. Expressed in 2011/12 prices, total expenditure on cancer-related research from 1970 to 2009 was £15 billion. Over the period 1991 to 2010, the interventions we prioritised in our study produced 5.9 million QALYs and a NMB of £124 billion, allowing for the net NHS costs resulting from them, and valuing a QALY at £25,000. The proportion of the benefit attributable to UK research was 17%. The lag between research funding and impact for cancer treatments was 15 years. Our best estimate of the health-gain IRR from UK cancer-related research was 10%, very similar to that of 9% for CVD research. The results suggest that, despite the uncertainties around the methods and estimates, the historical returns in terms of the NMB of the health gains derived in the UK from public and charitably funded biomedical and health research are substantial, and could by themselves justify the investment made.

## Endnotes

^a^We have used the term ‘interventions’ broadly throughout this paper to include treatments, screening programmes and a wide range of policies and information that have led to changes in smoking.

^b^NCRI members must have an annual cancer research spend in the UK in excess of £1 million, and have an appropriate peer-review system for ensuring the scientific quality of the research that they fund [57].

^c^Up until 2010, the Wellcome Trust had a policy not to fund cancer research. It changed its policy in recognition that the basic research it funded was increasingly having implications for our understanding of cancer.

^d^Data provided by the Higher Education Funding Council for England in personal correspondence.

^e^Data provided at our request by NATCANSAT produced from the national radiotherapy dataset for years 2009 to 2013.

^f^CWTS maintains a bibliometric database of all scientific publications (including health and biomedical research) for the period 1981 to 2013. This dataset is based on the journals and serials processed for the Internet versions of the Science Citation Index Expanded and associated citation indices, the Social Sciences Citation Index, and the Arts and Humanities Citation Index. This database is operated for bibliometric purposes in service contracts under a License Agreement with Thomson Reuters. See [58] for more information.

^g^We used HMG GDP Deflator [59] to estimate constant prices for 2011 (accessed 9 January 2013). We also compared the Biomedical Research and Development Price Index published by the National Institutes for Health Office of Budget ([60]; accessed 9 January 2013), and concluded that there was no material difference for the purpose of the current analysis.

## Abbreviations

CRUK: Cancer Research UK; CVD: cardiovascular disease; CWTS: Centre for Science and Technology Studies; DALY: Disability adjusted life year; DH: Department of Health; GDP: Gross Domestic Product; HSCIC: Health and Social Care Information Centre; IRR: the internal rate of return; MRC: Medical Research Council; NATCANSAT: National Clinical Analysis and Specialised Applications Team; NCRI: National Cancer Research Institute; NHMRC: National Health and Medical Research Council; NHS: National Health Service; NIC: Net Ingredient Cost; NICE: National Institute for Health and Clinical Excellence; NIHR: National Institute of Health Research; NMB: Net Monetary Benefit; ONS: Office of National Statistics; QALY: Quality adjusted life year; R&D: Research and Development; SIGN: Scottish Intercollegiate Guideline Network.

## Competing interests

The authors declare that there are no competing interests.

## Authors’ contributions

The project was conceived by JG and MB, and designed and executed by all the authors: JG led on the funding analysis; JG, AP, SG on the guidelines analysis; and MG and MB on the assessment of health gain and the calculation of the IRR. All authors were involved in synthesising and interpreting the results. All authors contributed drafts for various parts of the paper, critically reviewing various iterations and approving the final draft submitted.

## Supplementary Material

Additional file 1 Funding data. Click here for file

Additional file 2 Incidence and survival data. Click here for file

Additional file 3 Summary of interventions, their comparators, and sources, and health gain data. Click here for file

Additional file 4 Guidelines data. Click here for file
